# Retraction: NFIL3 facilitates neutrophil autophagy, neutrophil extracellular trap formation and inflammation during gout *via* REDD1-dependent mTOR inactivation

**DOI:** 10.3389/fmed.2023.1187721

**Published:** 2023-03-29

**Authors:** Frontiers Editorial Office

The authors contacted the Editorial Office to request the retraction of the cited article, stating that there were fundamental errors in the interpretation of Figures 4, 5. The authors also declared that they had discovered issues in the methodology of the study. An investigation was conducted in accordance with Frontiers' policies that confirmed that the conclusions reported in the article are no longer supported by the analyses; therefore, the article has been retracted.

This retraction was approved by the Chief Editors of Frontiers in Medicine and the Chief Executive Editor of Frontiers.

